# Gait disorders in adults and the elderly: A clinical guide

**DOI:** 10.1007/s00508-016-1096-4

**Published:** 2016-10-21

**Authors:** Walter Pirker, Regina Katzenschlager

**Affiliations:** 1 Department of Neurology, Medical University of Vienna, Währinger Gürtel 18–20, 1090 Vienna, Austria; 2 Department of Neurology, Wilhelminenspital, Vienna, Austria; 3 Department of Neurology and Karl Landsteiner Institute for Neuroimmunological and Neurodegenerative Conditions, Donauspital, Vienna, Austria

**Keywords:** Aging, Falls, Neurological gait disorders, Parkinsonism, Orthopaedic gait disorders

## Abstract

Human gait depends on a complex interplay of major parts of the nervous, musculoskeletal and cardiorespiratory systems. The individual gait pattern is influenced by age, personality, mood and sociocultural factors. The preferred walking speed in older adults is a sensitive marker of general health and survival. Safe walking requires intact cognition and executive control. Gait disorders lead to a loss of personal freedom, falls and injuries and result in a marked reduction in the quality of life. Acute onset of a gait disorder may indicate a cerebrovascular or other acute lesion in the nervous system but also systemic diseases or adverse effects of medication, in particular polypharmacy including sedatives. The prevalence of gait disorders increases from 10 % in people aged 60–69 years to more than 60 % in community dwelling subjects aged over 80 years. Sensory ataxia due to polyneuropathy, parkinsonism and frontal gait disorders due to subcortical vascular encephalopathy or disorders associated with dementia are among the most common neurological causes. Hip and knee osteoarthritis are common non-neurological causes of gait disorders. With advancing age the proportion of patients with multiple causes or combinations of neurological and non-neurological gait disorders increases. Thorough clinical observation of gait, taking a focused patient history and physical, neurological and orthopedic examinations are basic steps in the categorization of gait disorders and serve as a guide for ancillary investigations and therapeutic interventions. This clinically oriented review provides an overview on the phenotypic spectrum, work-up and treatment of gait disorders.

## Introduction

Walking is a common activity of daily living and at the same time a very complex one. It involves all levels of the nervous system and many parts of the musculoskeletal apparatus as well as the cardiorespiratory system. A person’s gait pattern is strongly influenced by age, personality and mood. Moreover, sociocultural factors play a role: for instance, persons living in large cities walk significantly faster than those living in rural areas [1]. The prevalence of gait and balance disorders markedly increases with age, from around 10 % between the ages of 60 and 69 years to more than 60 % in those over 80 years [2]. Gait impairments may greatly affect the quality of life [2] and restrict the personal independence of those affected. Moreover, balance and gait problems may be precursors of falls, which are the most common cause of severe injuries in the elderly [3]. Walking is a sensitive indicator of overall health status and the self-selected walking speed closely correlates with individual life expectancy in elderly persons [4]. Importantly, slow gait in elderly non-demented persons correlates more closely with the future emergence of dementia than subjective cognitive impairment [5, 6]. Nevertheless, gait disorders and falls are largely underdiagnosed and often receive inadequate evaluation [7]. Gait disorders are not specifically covered in most textbooks of neurology. Physicians are often not sufficiently trained to assess gait and axial motor symptoms are usually less well documented in medical reports than other parts of the neurological examination.

The causes of gait disorders include neurological conditions (e.g. sensory or motor impairments), orthopedic problems (e.g. osteoarthritis and skeletal deformities) and medical conditions (e.g. heart failure, respiratory insufficiency, peripheral arterial occlusive disease and obesity). In older age, gait disorders typically have several causes, which may include impaired proprioceptive function in polyneuropathy, poor vision, frontal gait disorder associated with vascular encephalopathy and osteoarthritis of the hips or knees. If a gait disorder has an acute onset, cerebrovascular, spinal and neuromuscular causes should be considered, as should adverse drug effects and psychiatric disorders. Possible medical causes include cardiorespiratory or metabolic disturbances and infections [8].

The evaluation of gait disorders includes the careful clinical observation of gait and a neurological and orthopedic examination based on the patient history, all of which guide the choice of ancillary diagnostic procedures if required and appropriate. This review is intended to be a guide for clinicians on the physiological basis of gait, the clinical examination and on typical causes of gait disorders.

## Physiological basis of gait

For normal gait all of the following functions and systems are required to be intact: locomotor function (for initiating and sustaining rhythmic gait), balance, postural reflexes, sensory function and sensorimotor integration, motor control, the musculoskeletal apparatus and cardiopulmonary functions. Afferent nerves from the visual, vestibular and proprioceptive systems provide essential information on the position of the body and its parts. Disturbances in one of these systems, e. g. proprioception, may be partially compensated by other sensory systems, such as vision. A centrally integrating system, which involves areas in the frontal cortex, the basal ganglia, the brain stem and the cerebellum, interprets the information received and selects the motor programs required for walking. The efferent system comprises descending pathways including the pyramidal tract, peripheral nerves, neuromuscular end plate and muscles. To some degree, rhythmic gait can also be sustained by spinal centers, resulting in spinal gait patterns in paraplegic patients while devices support them against gravity. In primates, brain stem centers have a central role in generating automatic walking, in particular the so-called midbrain locomotor center, which includes the pedunculopontine nucleus [9].

**Fig. 1:**
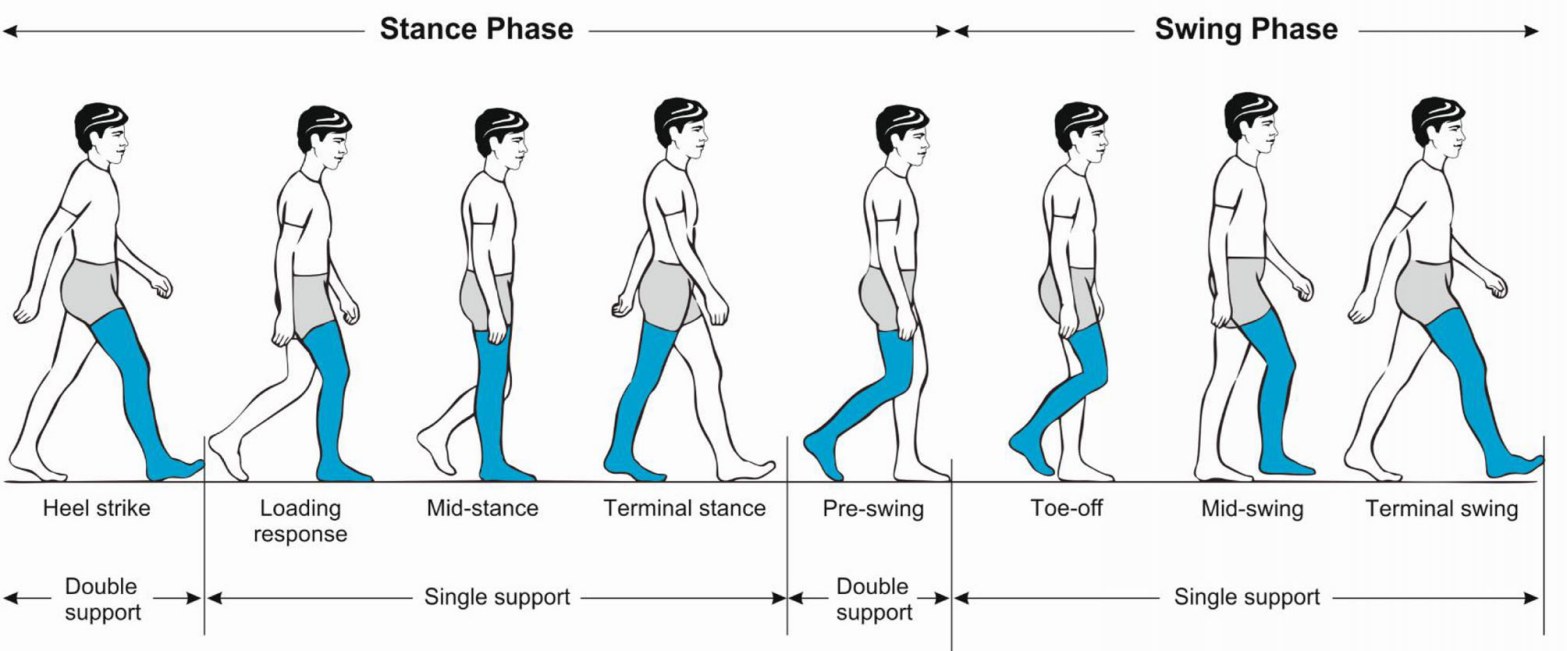
Phases of the normal gait cycle

Initiating gait requires a stable upright body position. Functioning postural reflexes are necessary to assume and sustain a stable body position. To start walking, one leg is raised and directed forward by flexing the hips and knee. Activation of the supporting contralateral leg and trunk muscles moves the body’s center of gravity over the weight-bearing leg and forward. The heel of the swinging leg is then placed on the ground. The body weight is gradually shifted to the sole and then onwards to the toes. During mid-stance, the opposite leg is lifted and moves forward until the heel strikes the ground. Meanwhile, the body is held upright, the shoulders and pelvis remain relatively level and each arm swings in the direction opposite to that of its ipsilateral leg. The gait cycle (Fig. 1) is divided into the stance and swing phase. The stance phase constitutes approximately 60 % of the gait cycle and is subdivided into initial contact (heel strike), loading response, mid-stance, terminal stance and pre-swing. Both feet are on the ground at the beginning and end of the stance phase. Each of these two double support periods lasts for approximately 10–12 % of the gait cycle. The swing phase takes up about 40 % of the gait cycle and is subdivided into initial swing (toe-off), mid-swing (tibia vertical) and terminal swing, terminated by the heel striking the ground [9, 10].

**Fig. 2:**
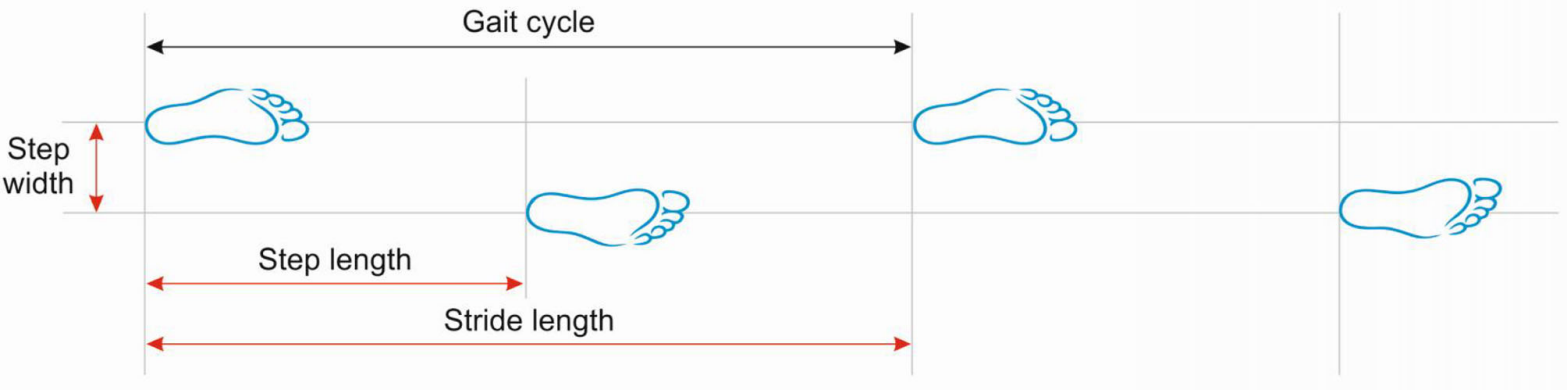
Basic terminology describing the gait cycle

Important measures of gait (Fig. 2) include walking speed, cadence (number of steps per unit of time), walking base width (measured from midpoint to midpoint of both heels), step length (measured from the point of foot contact to the point of contralateral foot contact) and stride length (linear distance covered by one gait cycle). The preferred walking speed in healthy adults up to the age of 59 years is approximately 1.4 m/s [11]. Average stride lengths in healthy adults range between 150 and 170 cm. The average cadence in young adults was reported to range between 115 and 120 steps/min. Ageing is associated with a decline in gait speed and step length whereas cadence remains relatively stable. Elderly subjects prefer a 40 % wider step width than young persons (average step width in elderly women approximately 8 cm and in elderly men 10 cm) [12].

## Role of cognition

Investigations over the past two decades have demonstrated the strong effects of cognition on gait [13], including the role of gait speed and gait disorders in older age as an indicator for the future development of dementia and of life expectancy [4–6]. Cognitive control is relevant for circumnavigating obstacles and for choosing the optimal route. Frontal executive functions, visuospatial perception and attention all contribute to walking safely. Psychological factors also influence gait. For instance, depression is associated with slower gait and anxiety may lead to an overly cautious gait. The role of cognition for gait is revealed in the multitask paradigm where persons are asked to perform mental tasks while walking. Elderly persons who stop walking while talking have a significantly higher risk of falling [14]. Further investigations have shown that patients with dementia walk slowly but in relation to their motor and cognitive deficits, they actually walk too fast leading to an increased risk of falling [15]. In situations where there is a risk of falling, healthy persons adopt a posture first strategy, which prioritizes the maintenance of balance over other tasks. This strategy is lost in patients with Parkinson’s disease [16]. These multiple interactions demonstrate that improving cognitive functioning may have an essential role in the rehabilitation of gait disorders.

## Clinical examination of gait

The clinical examination of gait provides a quick but integrative overview of the function of the structures involved in walking. It is important to observe the entire patient, from the front and from all sides, while walking over a distance of at least several meters without obstacles. If possible, walking should be observed without the subject wearing shoes. Normal walking appears rhythmical, flowing, effortless, with freely swinging legs and with an upright body posture. Normal walking is accompanied by movements of the head, the trunk and the arms (in the direction opposite to the movement of each leg). Table 1 summarizes the parameters that should be clinically examined, which include step length, stride length, step width, rhythm, speed, posture, swinging of arms and legs and the duration and type of contact with the floor [13].

**Table 1:** Parameters for the clinical examination of gait

Sitting unaided
Standing up from a sitting position (unaided and with/without use of upper limbs)
Posture (trunk, neck and head, upright, bent or asymmetrical)
Stance (narrow/wide base)
Gait initiation (blockage)
Walking (smooth, stiff, insecure, symmetrical, limping)
Step length, lifting of feet, contact with ground, wide/narrow base
Speed
Arm swing
Freezing
Turning
Postural reflexes (pull or push test)
Sitting down (“motor recklessness”)
**Complex tests of stance and gait**
Tandem stance
Tandem gait
Romberg’s test (standing with eyes closed and narrow base)
Blind gait
Walking backwards
Walking fast
Walking slowly (in a deliberate manner)
Running
Turning quickly
Turning on the spot
Unterberger’s test (walking on the spot with eyes closed)
Standing and walking on heels
Standing and walking on toes
Hopping on one foot
Dual task maneuver (walking while talking or carrying objects)
Functional reach

Semiquantitative methods of gait assessment may be applied for diagnostic purposes (e. g. spinal tap test when normal pressure hydrocephalus is suspected) and to evaluate therapies. Examples include measuring the freely chosen and the maximum speed over a defined distance, such as 10 m using a stop watch. The number of steps taken over that distance can also be counted [17]. The timed up and go (TUG) test is a simple assessment developed to evaluate the risk of falling in geriatric patients: measured as the time that a patient requires to stand up from a chair with arm rests, walk 3 m (using usual walking aids if necessary), turn around, walk back and sit back down [18].

A general neurological assessment should always follow the evaluation of gait. Nearly all components of the clinical neurological examination may provide additional information, which may help in the classification of a gait disorder. It is advisable to perform the examination with most of the patient’s clothes removed, if possible. This also helps with the evaluation of any orthopedic abnormalities. The patient should be inspected from all sides while standing still. This allows detection of asymmetries, postural abnormalities, differences in leg length and axial or other deformities. Orthopedic disturbances are frequently associated with asymmetrical gait patterns (limping). In some cases, ophthalmological and medical examinations are useful to assess cardiorespiratory function and orthostatic blood pressure regulation or to perform angiological tests. An otological or neuro-otological assessment may be required if a vestibular cause of a gait disorder is suspected. The subject history must include questions on any neurological, orthopedic and medical symptoms or prior history, patient medication and detailed questioning with respect to any falls. Maximum walking distance, the number of rests needed to cover that distance, limiting factors, such as pain or shortness of breath as well as the use of walking aids should be documented.

## History and classification of falls

In elderly persons, pre-existing difficulties with walking and balance are more commonly the cause of falls than acute disturbances, such as syncope, seizures or stroke [19]. Almost one third of all persons over the age of 65 years fall every year and more than half of those fall more than once. Approximately 10–15 % of these falls lead to serious injuries, such as traumatic brain injury or hip fractures. It has been estimated that inadvertent injuries are the fifth most common cause of death in elderly persons [3]. Persons at risk should be questioned about the type and frequency of falls and any symptoms prior to or following falls. Factors that might precipitate falls in the home include loose carpets on wooden floors. A precise history of the patient’s medication is essential, in particular with respect to drugs with sedating or blood pressure lowering effects, such as tricyclic antidepressants, sedatives, anxiolytics, neuroleptics and antihypertensive drugs [13, 20]. Polypharmacy is regarded as an important risk factor for falls in the elderly; however, newer studies suggest that polypharmacy only poses a risk if it includes medications that increase the risk for falls, such as sedatives, antidepressants and benzodiazepines [21, 22]. Table 2 shows a phenomenological classification of falls, which may assist in the etiological classification. Table 3 summarizes relevant risk factors for falls and Table 4 shows general measures recommended for the prevention of falls and fall-related injuries. For further reading on the topic of falls we refer to the excellent reviews by Bloem and his group [20, 23, 24].

**Table 2:** Classification of fall syndromes (modified from Nutt [31])

Type	Causes
**Collapsing**	
– Atonic seizure, negative myoclonus, cataplexy	
– Syncope	Orthostatic hypotension and others
**Tonic** (“falling like a log”)	
– While standing	PSP, thalamic astasia, tonic seizure
– On changing posture/position	Parkinson’s disease (PD)
**Tripping**	Weak foot extensors, spasticity, PD
**Freezing**	PD, frontal gait disorder
**No specific pattern**	Attention deficit, dementia

*PSP* progressive supranuclear palsy; *PD* Parkinson’s disease

**Table 3:** Risk factors for falls

Female gender, low body weight, age >80 years	
Number of falls in previous year/month	
Use of sedatives, particularly with long half-life	
Limited physical activity	
Difficulties rising from sitting position	
Reduced muscle strength in the lower limbs	
Impaired balance	Standing
	Walking
	Turning
Impaired postural reflexes	
Impaired vision	
Impaired cognitive functions, depression, anxiety

**Table 4:** General measures to prevent falls and fall-related injuries

Check entire list of medication
Avoid sedatives, particularly with long half-life
Avoid (classical) neuroleptics and tricyclic antidepressants
Check the indications for and dose of atypical neuroleptics
Increase physical activity
Healthy diet, avoid malnutrition and overweight
Muscle training
Balance training
Anxiolytic and antidepressant therapy
Behavioral therapy for anxiety, depression and dementia
Therapy of orthostatic hypotension
Treatment for osteoporosis
Adequate footwear
Protective devices such as hip protectors
Remove risks at home and adjust personal environment
Electronic warning systems

## Protective strategies and age-related changes of gait

Even in healthy persons, any suspected or actual threat to balance induces changes in the strategies for standing and walking: the stance and gait base is widened, bipedal floor contact is prolonged, step length becomes shorter, the feet are lifted less high during the swing phase, walking becomes slower and the posture becomes stooped. A typical example where this may occur in healthy persons is walking on icy ground or on a slippery floor [9]. The fear of falling and the actual risk of falling increase with age. Older persons are therefore more likely to use these protective gait strategies. As muscle power diminishes and proprioception and vision become impaired with age, body sway on standing, which is constantly present to a slight degree, increases. In younger persons this sway can be compensated by activating the muscle groups around the upper ankle joints. Older persons shift this compensation to the proximal muscle groups around the hips due to loss of distal proprioception. This requires an increased reliance on vestibular afferents, which undergo less change during the ageing process.

The preferred walking speed in apparently healthy elderly subjects declines by 1 % per year from a mean of 1.3 m/s in the seventh decade to a mean of 0.95 m/s in those aged over 80 years [11]. The decline in walking speed is caused by a decrease in step length rather than by a change in cadence [25]. Although these gait changes are to some degree a consequence of normal ageing, individual walking speed in elderly subjects is a strong indicator of general health and survival [4].

## Epidemiology and classification of gait disorders

The Bruneck study, in which a representative sample of the population of a small region in northern Italy is being followed longitudinally, has provided valuable epidemiological data on various gait disorders in 488 persons: between the ages of 60 and 97 years one third of this population had a gait disorder, with a marked increase in prevalence with age, between the ages of 60 and 69 years, the prevalence was 10 % and in those over 80 years it was >60 % [2]. In two thirds of those affected by any gait disorder, the cause was neurological and in approximately one half, the cause was non-neurological, indicating that there was a considerable overlap of patients affected by neurological as well as by non-neurological gait disorders. Among the neurological causes, sensory ataxia (18 %) and parkinsonian (16 %) gait disorders were the most common, followed by frontal (8 %), cerebellar ataxic gait disorders, cautious gait and hypotonic paretic, spastic, vestibular and dyskinetic gait disorders. In approximately one third of the patients the gait disorder was due to more than one neurological cause, making a precise classification difficult [2]. It has to be pointed out that these prevalence rates refer to elderly people living in the community. Substantially higher rates of gait disorders are to be expected among geriatric hospital and nursing home residents [3].

**Fig. 3:**
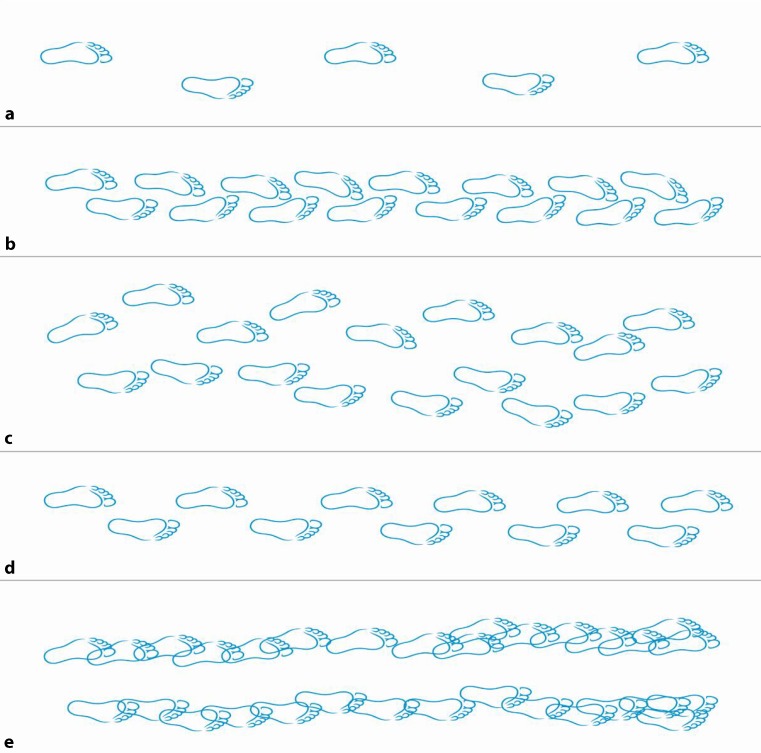
Graphic representation of the step sequence in classical gait disorders. **a** normal gait, **b** spastic paraparetic gait, **c** cerebellar ataxic gait, **d** parkinsonian gait and **e** frontal gait. Note narrow step width and inwards rotation in paraspastic gait, broadened base and marked irregularity in cerebellar gait, shortened and mildly irregular step length in parkinsonian gait and broad-based, short-stepped, irregular walking in frontal gait disorder

Table 5 shows a phenomenological classification of common gait disorders. Here we use a more hierarchical, clinicoanatomical classification and differentiate gait disorders due to musculoskeletal (orthopedic), neuromuscular (peripheral neurological), spinal and brain disorders. Fig. 3 features a graphic representation of the step sequence in normal gait and in some important gait disorders.

**Table 5:** Phenomenological classification of gait disorders (modified from Ružička and Jankovic [9])

Gait disorder	Characteristics
Hemispastic gait	Unilateral extension and circumduction
Paraspastic gait	Bilateral extension and adduction, stiff
Ataxic gait	Broad base, lack of coordination
Sensory ataxic gait	Cautious, worsening without visual input
Cautious gait	Broad based, cautious, slow, anxious
Freezing gait	Blockage, e. g. on turning
Propulsive gait	Centre of gravity in front of body, festination
Astasia	Primary impairment of stance/balance
Dystonic gait	Abnormal posture of foot/leg
Choreatic gait	Irregular, dance-like, broad-based
Steppage gait	Weakness of foot extensors
Waddling gait	Broad-based, swaying, drop of swinging leg
Antalgic gait	Shortened stance phase on affected side
Vertiginous gait	Insecure, tendency to fall to one side
Psychogenic gait disorder	Bizarre, rarely falls

## Musculoskeletal gait disorders

Osteoarthritis and skeletal deformities of the lower extremities are the most common reasons for non-neurological gait disorders in adults [2]. The resulting orthopedic gait disturbances may be characterized by a limited range of motion, avoidance of weight-bearing and asymmetry or limping.

### Antalgic gait

To avoid pain weight is put on the affected leg for as short a time as possible, resulting in a limp. The patients appear to be walking as if there were a thorn in the sole of the foot. To reduce the load on the affected leg the patients lift and lower their foot in a fixed ankle position. Walking aids, such as crutches are carried on the unaffected side in order to shift weight away from the affected side and to the upper extremity. Typical causes are painful conditions in the lower extremities, such as knee osteoarthritis, ankle sprains and stress fractures of the foot [10].

### Coxalgic gait

In patients with hip pain the upper trunk is typically shifted towards the affected side during the stance phase on the affected leg. This is an unconscious adaptive maneuver which reduces the forces exerted on the affected hip during the stance phase. In contrast to the Duchenne sign as a consequence of gluteus medius muscle weakness (see waddling gait), the contralateral hemipelvis does not drop but remains level during the stance phase of the affected side [10].

### Knee hyperextension gait

Quadriceps muscle weakness results in knee hyperextension during the early stance phase. Initial contact may occur with a flat foot. The knee is stabilized by the posterior ligaments. Increased ankle plantar flexion and hip extension serve to extend and advance the affected leg during the stance phase [10].

### Other causes of limping gait

Other important reasons for limping are deformities and contractures of the hip, knee and leg length discrepancies. When there are differences in leg length, the body moves slightly up and down with each step, the head and trunk are bent towards the affected side, the shoulder on the affected side is held higher and the arm on the contralateral side swings at a greater distance from the body than on the affected side. Patients may show compensatory foot plantar flexion in the shorter limb or habitual hip and knee flexion in the longer leg. Spinal pain produces an inhibited, slow, short-stepped gait and decreased lumbar lordosis. Patients typically avoid heel strike during the early stance phase. Kyphosis and ankylosing spondylitis result in a stooped posture and truncal immobility and can mimic postural abnormalities in Parkinson’s disease (PD) [8]. For other orthopedic gait disorders we refer to the excellent review by Lim et al. [10].

## Neuromuscular and myelopathic gait disorders

Peripheral paresis that is severe enough to cause a gait disorder can usually be detected on standard clinical neurological examination.

### Waddling gait

Weakness of the hip girdle and upper thigh muscles, for instance in myopathies, leads to an instability of the pelvis on standing and walking. If the muscles extending the hip joint are affected, the posture in that joint becomes flexed and lumbar lordosis increases. The patients usually have difficulties standing up from a sitting position. Due to weakness in the gluteus medius muscle, the hip on the side of the swinging leg drops with each step (referred to as Trendelenburg sign) [10]. The gait appears waddling. The patients frequently attempt to counteract the dropping of the hip on the swinging side by bending the trunk towards the side which is in the stance phase (in the German language literature this is referred to as Duchenne sign) [26]. Similar gait patterns can be caused by orthopedic conditions when the origin and the insertion site of the gluteus medius muscle are closer to each other than normal, for instance due to a posttraumatic elevation of the trochanter or pseudarthrosis of the femoral neck [10].

### Steppage gait

When the muscles that lift the foot are paretic the patient must lift the leg higher than usual during the swing phase. The resulting gait pattern is referred to as steppage gait. Patients are unable to stand or walk on their heels. Peroneal splints and orthopedic footwear are usually helpful. Patients with pareses of the plantar flexors muscles cannot flex the foot at the end of the stance phase. They cannot perform toe walking [9].

### Neurogenic and intermittent claudication

Intermittent claudication is a symptom of peripheral arterial occlusive disease. After having walked over a distance which is individually characteristic, the patients experience pain or cramps in the calves, feet or thighs which typically subsides on standing still.

#### Lumbar spinal stenosis and neurogenic claudication

**Fig. 4:**
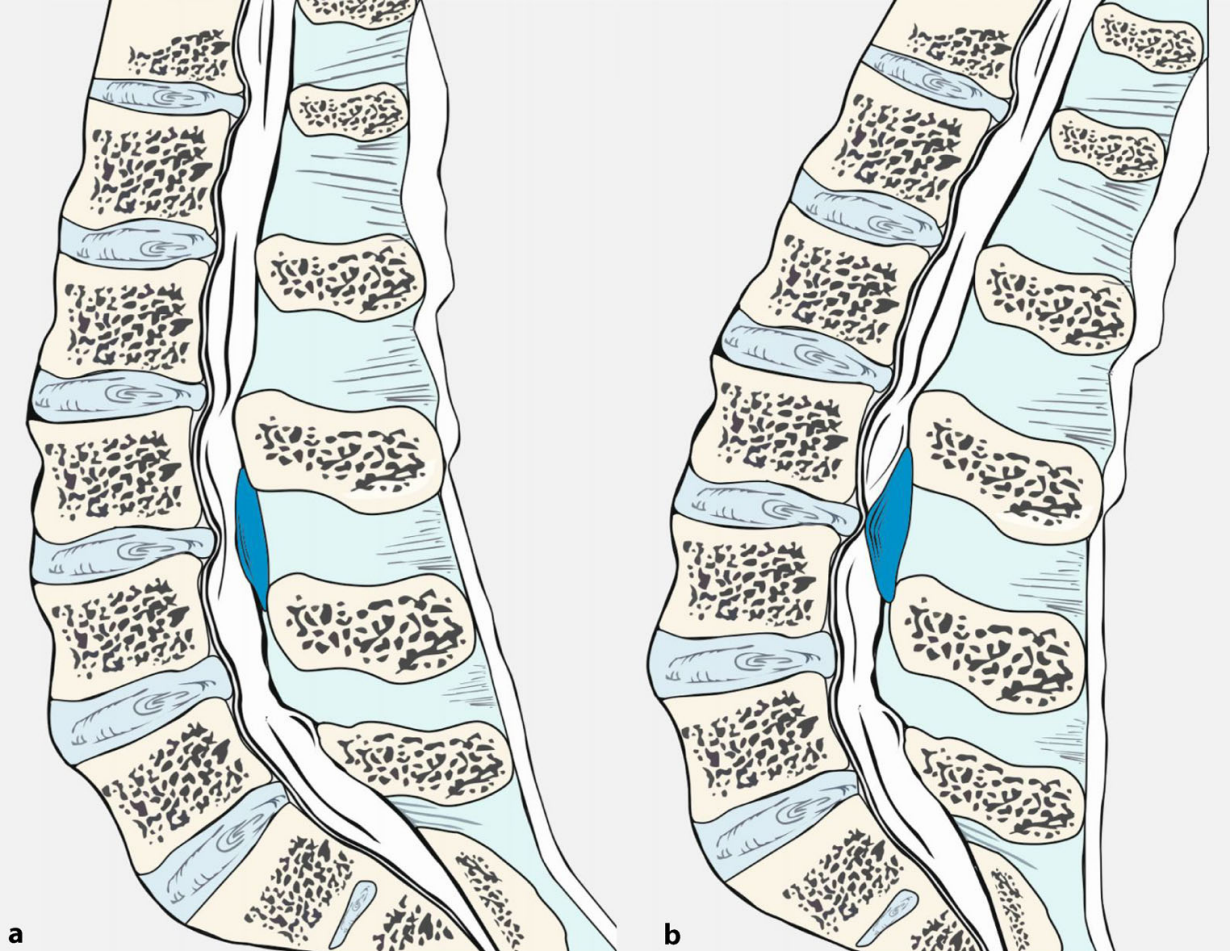
Pathophysiology of cauda equina compression in lumbar spinal stenosis. Flexed lumbar spine (**a**) as in the normal sitting position. Extension of the spine (**b**) as during normal walking or during the hyperextension maneuver leads to thickening of the ligamentum flavum and a decrease in the gap between the posterior margin of the intervertebral disc and the facet joints, both resulting in a reduction of the diameter of the spinal canal and dural sac

Lumbar spinal stenoses may induce symptoms following an individually typical latency on standing or when walking due to swelling of the cauda equina, which leads to compression. This is referred to as neurogenic claudication. The symptoms of lumbar spinal stenosis can be explained by an increase in lumbar lordosis and spinal canal stenosis in an upright position compared to the sitting position (Fig. 4, [27]) or if spondylolisthesis is present by a shift of the vertebrae while standing and walking. Following an individually characteristic distance, walking becomes associated with deep muscular pain and with neurological deficits, such as sensory deficits and paresis in the lower limbs, which resolve within minutes when the affected person sits or lies down. Activities performed in a flexed posture, such as cycling often cause less problems than walking. For the same reason, walking uphill may be tolerated better than walking downhill. Clinical neurological examination at rest may be entirely normal but there is usually pain on hyperextension of the lumbar spine [10].

Non-surgical treatment includes training how to lift the pelvis while standing and walking or corsets to counteract lordosis. Frequently these measures are of limited value and often surgical decompression is the only effective treatment [28].

### Myelopathic gait

Cervical spondylotic myelopathy is a relatively common cause of gait disturbance in the elderly [3]. Degenerative osteophytes and ligamentous hypertrophy lead to narrowing of the spinal canal and mechanical compression of the cervical spinal cord. Gait and balance problems are the main clinical manifestations. The gait is stiff and paraparetic spastic but may also be spastic ataxic due to dorsal column dysfunction. Cervical pain, upper limb sensory symptoms and loss of dexterity are common but may be absent in a minority of affected patients. In severe cases, urinary urgency may occur. Compressive cervical myelopathy tends to be progressive and surgical decompression should be considered in symptomatic cases [29].

## Gait disorders associated with brain dysfunction

This category encompasses all gait disorders related to brain disease or dysfunction, i. e. the neurological gait disorders in a narrower sense. It largely overlaps with the categories of middle and higher level gait disorders in the classification proposed by Nutt et al. [30–32]; however, some middle level gait disorders, such as spastic gait may also be caused by spinal lesions.

### Cautious gait

Cautious gait (sometimes termed senile gait) refers to an excessive degree of age-related changes in walking and fear of falling. The walking difficulties seem out of proportion when considering the patient’s actual sensory or motor deficits. The gait appears slow, with a wider base than normal, reduced arm swing bilaterally and a slightly stooped posture. This type of gait change often occurs after the first time a patient has fallen. Without treatment, excessively cautious gait may lead to considerable handicap. Phobic gait disorder can be considered the maximum variant of cautious gait: these patients suffer from excessive fear of falling of which they are aware and which may result in a complete inability to walk. Gait may improve even with very slight support, such as another person touching the patient’s hand. Some patients respond to gait and balance training and anxiolytics [9].

### Spastic gait disorders

#### Spastic hemiparetic gait

Spastic hemiparesis is characterized by a dominance of the tonus in the upper limb flexor muscles: the arm is held in an adducted posture and is bent and rotated inwards, the forearm is pronated and the hand and the fingers are flexed. The leg is slightly bent at the hip, the knee cannot be extended fully at the end of the stance phase and the foot is inverted and in a plantar flexed position. Gait is slow, with a wide base and asymmetrical with a shortened weight-bearing phase on the paretic side. During the swing phase, the paretic leg performs a lateral movement (circumduction) which is characteristic of this gait disorder, also termed Wernicke-Mann gait. Spastic gait problems typically worsen on attempts to walk faster. Treatment mainly consists of physiotherapy although in some cases local botulinum toxin injections may be helpful.

#### Spastic paraparetic gait

In paraspastic gait, the legs are usually slightly bent at the hip and in an adducted position. The knees are extended or slightly bent and the feet are in a plantar flexion position. This posture requires circumduction of the legs during walking. The gait may appear stiff (spastic gait disorder) or stiff as well as insecure (spastic ataxic gait disorder). In spastic paraparetic gait, each leg appears to be dragged forward. If the muscle tone in the adductors is marked, the resulting gait disorder is referred to as scissor gait. Treatment options include physiotherapy and medication with a muscle relaxing effect, such as baclofen and tizanidine. In selected cases, botulinum toxin injections may help alleviate spasticity and improve motor function [9].

### Ataxic gait disorders

#### Cerebellar ataxic gait

Cerebellar ataxia and sensory ataxia can be distinguished based on clinical characteristics. In sensory ataxia, the loss of proprioception may partially be compensated by visual input. In contrast, this is not the case in cerebellar ataxia; therefore, standing still with a narrow stance base (Romberg’s test) and walking with closed eyes lead to a worsening of imbalance in sensory ataxia, whereas these tests reveal less difference in cerebellar ataxia. In cerebellar ataxia, stance and gait appear broad based, insecure and wobbly. Leg movements and step length are irregular and variable. The ataxia increases on turning and during complex gait tests, such as tandem walk and walking on uneven surfaces. Gait initiation is usually normal. The patients attempt to compensate lateral body sway by walking cautiously, stooping slightly and steadying the stance foot by bending at the hip. The causes of cerebellar disease may be vascular, toxic (e. g. alcohol), degenerative (e. g. hereditary cerebellar ataxias), inflammatory (e. g. multiple sclerosis) or neoplastic. The pathomechanism includes disturbances of balance and motor coordination. Studies in humans with cerebellar lesions have convincingly demonstrated that cerebellar gait ataxia is more closely related to balance than to leg placement deficits [33]. Control of balance is predominantly localized in the vermis region of the cerebellum. The intermediary zone or paravermal structures are important for the control of the timing and amplitude of targeted limb movements; however, the cerebellar hemispheres are also involved in the control of bipedal human gait [34]. Unilateral lesions of the cerebellar hemispheres induce ipsilateral limb ataxia. Isolated lesions of the vermis or the paravermal structures may cause gait ataxia without affecting the limbs. In some patients with cerebellar disease, postural innervation is associated with slow oscillations of the entire body or the head (titubation).

#### Sensory ataxic gait

Disturbances of proprioceptive function may occur in sensory polyneuropathy or dorsal column lesions. In sensory ataxia, stance and gait appear broad based and insecure. The step length is shortened. Gait is slower and more cautious compared to cerebellar ataxia. The feet are sometimes lifted high and gait may have a stomping quality. The patients use visual control to compensate for the loss of proprioception. Consequently, loss of visual function causes marked worsening of ataxia. This may become evident during clinical testing (Romberg’s test, walking with closed eyes), walking in darkness or a sudden disturbance of vision (e. g. due to acute ischemic optic neuropathy if the contralateral eye has pre-existing impaired vision). Ataxic gait and the associated risk of falling are also worsened if circumstances require a slower gait, e. g. unknown territory or obstacles [35]. As in cerebellar ataxia, complex clinical tests including tandem walk and walking on uneven surfaces increase insecurity in sensory ataxic gait. Some hereditary ataxias may cause both cerebellar and sensory type ataxia due to a combination of cerebellar disease and sensory neuropathy or a dorsal column lesion. Examples include spinocerebellar ataxias that are associated with polyneuropathy. Treatment should target the underlying cause if possible. Physiotherapy including gait and balance training should be offered although the effect of this approach is often limited. Walking aids appropriate for each disease stage need to be considered.

### Frontal gait disorder/higher level gait disorders

Frontal gait disorders are common in older age. They may lead to a misdiagnosis of Parkinson’s disease (PD), particularly if tremor (of any cause) is also present. These gait problems may however also occur in younger patients who have frontal lesions. Various terms for this type of gait disorder have been used in the literature, including frontal gait ataxia, gait apraxia, magnetic apraxia, lower body parkinsonism and marche à petits pas, all of which describe the same clinical syndrome [9].

Nutt, Marsden and Thompson coined the terms “highest” or “higher level gait disorder” in 1993 [30]. This comprises all gait or balance problems which are not explained by peripheral (motor and sensory), pyramidal, cerebellar of basal ganglia lesions. This group of gait disorders includes frontal as well as senile gait disorder (cautious gait), subcortical and frontal balance impairment (subcortical/frontal disequilibrium) and isolated gait ignition failure. Magnetic resonance imaging (MRI) studies have shown that this group of gait disorders may be caused by frontal as well as parietal lesions [36]. Further causes include lesions in the corpus callosum, which may induce gait problems by disrupting interhemispheric communication [37]. A recent imaging study suggested that balance and gait problems in patients with higher level gait disorders are associated with grey matter atrophy in the midbrain and caused by dysfunction in a network linking the primary motor cortex with the midbrain locomotor region [38]. These novel clinico-anatomical findings seem to justify the use of the term “higher level gait disorders”, although this concept has been criticized by some authors for its complexity [25, 39]. In clinical practice the term “frontal gait disorder” remains in common use. Common causes of frontal gait disorders include vascular lesions (e.g. white matter and basal ganglia lacunar infarction and territorial infarction in the anterior cerebral artery territory). In normal pressure hydrocephalus, clinical problems which may emerge in association with the typical gait disorder are bladder problems and cognitive deficits. Further causes include advanced Alzheimer’s disease, frontotemporal lobar degeneration and space occupying lesions of the frontal lobe. Patients with frontal gait disorders frequently appear to have forgotten how to perform the act of walking. They have difficulties standing up, inadequately adapt the posture when changing position (for instance, they may extend rather than bend the trunk and legs on attempting to stand up) and have difficulties achieving a stable position. The gait has a broad base, step length is short and they appear anxious. The arms may be extended laterally and arm swing may be reduced. Trunk posture may be stooped, upright or even hyperextended. Inhibition of gait initiation is common and is the only symptom in isolated gait ignition failure. Some patients attempt to initiate gait by swaying the trunk laterally or performing exaggerated arm movements. Walking appears to present great difficulty and is shuffling, the feet may appear glued to the ground (“magnetic feet”). In other cases, patients seem to be scuffling their feet. After having walked a few meters, gait often improves. Blocked gait or freezing episodes may occur, particularly on turning and when faced with obstacles such as thresholds. Balance and postural stability are impaired. In some patients, retropulsion occurs which may lead to backward falls.

There is a marked discrepancy between the severity of a patient’s gait difficulties and the moderate impairment in a sitting or supine position. Many patients are capable of performing regular, rapid stepping movements while in a sitting position. Conversely, mild rigidity, gegenhalten and bradykinesia are commonly found, predominantly in the lower limbs, hence the term lower body parkinsonism [40]. Depending on the etiology, patients may have increased deep tendon reflexes or positive pyramidal tract signs, signs of pseudobulbar palsy (e.g. dysarthria, dysphagia), frontal release signs (e.g. snout or grasp reflex) or cognitive changes including dementia. In some cases, causal treatment is available, such as shunting in normal pressure hydrocephalus and treatment of vascular risk factors; however, in most cases physiotherapy including gait training is the only option, often with limited permanent effects. Rhythmic acoustic or visual signals, which may be employed in PD are rarely effective. There is no evidence of efficacy of antiparkinsonian drugs, including levodopa, although they may be worth trying in cases with clinical parkinsonism.

### Parkinsonian gait (rigid akinetic gait disorder)

The cardinal motor signs of PD are bradykinesia, rigidity, rest tremor and impaired postural stability. In the majority of patients, the symptoms initially affect only one side of the body and spread to the other side. Even at very early stages of the illness, walking frequently appears somewhat slow. In hemiparkinsonism, physiological arm swing is reduced and the leg may be slightly dragged on the affected side. As the disease progresses, the typical rigid akinetic gait impairment develops, which includes slow gait with a short step length, a narrow base and a stooped posture involving neck, shoulders and trunk. Arm swing is reduced and in more advanced stages the arms are held in an adducted and bent position. The feet are lifted less high than normally, which may lead to shuffling gait. The step to step variability of the gait cycle increases. When asked to walk faster, patients increase the step frequency rather than step length. Performing other tasks simultaneously, such as walking while talking, worsens the gait. Commonly, patients with PD find it easier to climb up stairs than to walk on a level surface. Standing up from a sitting position is impaired, which becomes apparent when patients are asked to rise without using the arms. Many patients develop a propensity to lean forward while walking, associated with increased step frequency, reduced stride length and bent truncal posture. This particular gait pattern in PD is referred to as festination and carries a risk of forward falls. Impaired postural reflexes are a dominant cause of falls in PD and are apparent on performing the pull or push test. Changing position becomes difficult as axial bradykinesia increases. Patients turn en bloc using many small steps.

#### Freezing

Difficulties with gait initiation and freezing typically occur on turning or when approaching obstacles or narrow passages such as doors. In some PD patients freezing develops at a very early stage and resolves after starting antiparkinsonian medication. In PD patients with motor fluctuations freezing is usually linked to periods with loss of levodopa response (OFF phases). As the disease progresses, however, freezing may become resistant to levodopa and may occur during periods of otherwise good levodopa response (ON phases). Rarely, freezing may improve overnight as the effect of dopaminergic medication wears off, suggesting a causal role of dopaminergic drugs [41]. There are three phenomenologically distinct types of freezing: the purely akinetic form is rare and is characterized by start failure or a complete stop while walking, the second type involves shuffling on the spot and the third an insufficient shuffling with very small steps [42].

#### Management of parkinsonian gait difficulties

The gait disorder and to a lesser degree the postural instability of PD often respond to antiparkinsonian medication; however, levodopa and particularly dopamine agonists may contribute to an existing orthostatic hypotension and thereby may make walking more insecure. Management includes modification of the antiparkinsonian medication, medical and non-medical treatment of orthostatic hypotension, cholinesterase inhibitors in patients with cognitive impairment and physiotherapy. Walking aids, such as 4‑wheel walkers with brakes, may be helpful. In freezing, improvements in overcoming the blockages can sometimes be achieved by using rhythmical acoustic signals (e.g. counting and clapping) or visual impulses (e.g. horizontal stripes attached to or painted on the floor in spots where freezing typically occurs at home or holding a walking stick upside down in front of the foot). A new technique is the use of a stick projecting a laser line on the floor to step over [43]. These tricks tend to be more effective while they represent novel stimuli. Cognitive strategies may also be helpful, such as directing one’s attention to each individual step rather than the act of walking as such, consciously attempting to make individual large steps or mentally counting [41]. Surgery for PD (mainly deep brain stimulation) usually improves only those aspects of gait and posture that are associated with motor fluctuations and dyskinesia and which are responsive to levodopa.

#### Other conditions associated with rigid akinetic gait disorder

In PD a marked rigid akinetic gait disorder with clinically relevant postural instability usually occurs in advanced disease stages. If these problems present early into the disease course or on presentation, the diagnosis should be carefully re-evaluated. In these cases, possible causes include old age or a longer disease duration than realized by the patients, e. g. if they attributed the general slowing to the ageing process. Comorbidities may be present, such as vascular encephalopathy, sensory ataxic gait disorder due to polyneuropathy or spasticity due to cervical myelopathy. Other differential diagnoses include frontal gait and secondary parkinsonian syndromes, in particular vascular parkinsonism. The atypical parkinsonian syndromes, such as multiple system atrophy (MSA) and progressive supranuclear palsy (PSP) typically lead to early problems with balance and gait. These neurodegenerative conditions respond less well to dopaminergic therapy than PD and have a more rapid disease course.

*Multiple system atrophy (MSA).* Patients with MSA often have combined parkinsonism and cerebellar signs, resulting in a gait disorder that is rigid akinetic as well as ataxic. Orthostatic dysregulation may contribute to the difficulties in walking, even in early disease stages. Postural abnormalities in MSA may be severe and may further impair gait. These include a marked forward flexion of the neck (disproportionate antecollis), and a forward or pronounced lateral flexion of the trunk termed Pisa syndrome. Camptocormia is a massive forward flexion of the trunk on standing or sitting which completely resolves in a supine position. Camptocormia may also occur in PD.

*Progressive supranuclear palsy (PSP).* Patients with PSP typically fall during the first year of the illness and this frequently happens backwards. Posture tends to be upright rather than bent forward and the neck may be hyperextended (retrocollis). Gait may be rigid akinetic as in PD but is more typically broad based. Many patients with PSP tend to fling the legs forward in an uncontrolled manner while walking and to turn around abruptly. Similarly, sitting down is typically an abrupt movement and appears as if the patients would let themselves fall onto the chair. These changes in motor control have been termed motor recklessness. The cardinal symptoms of the classical clinical type of PSP (also termed Richardson syndrome) are predominantly axial parkinsonism and restrictions in vertical gaze range. Another relevant clinical manifestation is pure akinesia with freezing gait, predominantly with gait impairment and freezing [44].

Patients with atypical parkinsonian syndromes should primarily receive levodopa and if well-tolerated, high doses should be tried. Response is usually modest in MSA and poor in PSP. Physiotherapy should include gait training and the adaptation of appropriate walking aids and protection from falls.

### Dystonic gait disorder

Primary generalized dystonias have a usual onset in childhood and in early adulthood. Secondary dystonias affecting gait may also occur in adulthood and include toxic or hypoxic basal ganglia damage and tardive dystonia following long-term treatment with dopamine receptor blocking drugs. Moreover, PD may be associated with dystonic OFF symptoms as well as dystonia as part of levodopa-induced dyskinesia.

Dystonic gait disorders frequently appear bizarre, particularly because activity increases dystonic tonus and posture. The abnormal posture of the foot in dystonic gait typically involves inversion, plantar flexion and tonic extension of the big toe. In many patients complex types of walking, such as walking backwards and running are paradoxically less impaired than walking forward and may seem completely unaffected. Sensory tricks, e. g. resting one’s hand on one’s neck, may improve or even normalize dystonic gait in some patients, all of which may lead to confusion with a psychogenic gait disorder.

### Choreatic gait disorder

Huntington’s disease has an autosomal dominant inheritance pattern and may become manifest at any age. It is characterized by cognitive decline and psychiatric disturbance and the choreatic movement disorder also affects the patients’ gait. Choreatic gait disorders may also occur in levodopa-induced dyskinesia in PD, in tardive dyskinesia and, less frequently, with hypoxic lesions in the basal ganglia, e. g. following cardiopulmonary bypass surgery (“post pump” chorea). Typical choreatic gait is impaired by sudden involuntary movements affecting knee and hip flexion and it appears irregular, dance-like and swaying. There is marked variability in step length and direction. To compensate for these intrusive movements, patients widen the stance base and walk more slowly. Falls typically occur only in severe cases. Tardive dyskinesia and levodopa-induced dyskinesia may appear stereotypical or bizarre.

### Myoclonic gait disorder

Myoclonus consist of short-lasting, involuntary jerks which result in the movement of a joint (positive myoclonus) or with sudden loss of muscle tone (negative myoclonus). Myoclonus of the trunk and the lower limbs may cause gait difficulties and insecurity on standing up, sudden giving way of the hips and knees and falls. A typical cause of this clinical syndrome in older age groups is generalized cerebral ischemia or hypoxia.

### Orthostatic tremor

Orthostatic tremor is a rare neurological disorder which may greatly affect the patients’ quality of life and may result in marked impairment. Its cause remains unidentified. While sitting or lying down, patients have entirely normal neurological findings. Once patients stand up, however, a tremor of very high frequency (13–20 Hz) sets in, which is perceived as an inability to stand and as fear of falling, rather than actual tremor and which is barely recognizable on inspection due to the high frequency. It mainly affects the muscles of the lower extremities and the trunk and affects standing much more than walking. Falls occur but are relatively rare in relation to the patients’ fear of falling. These characteristics frequently lead to confusion with a functional disorder, with a long average latency period between disease onset and the correct diagnosis, which further heightens patient distress. Clonazepam shows at least mild benefits in the majority of patients. Patients with orthostatic tremor may also benefit from propranolol or gabapentin [45].

### Subcortical disequilibrium (thalamic astasia)

Thalamic astasia typically occurs following unilateral lesions in the thalamus (ventrolateral or posterior nucleus) or in the lenticular nucleus. Infarctions and hemorrhages in these areas lead to a tendency to fall backwards or to the contralateral side while sitting or standing, including in patients who have no relevant motor deficits. Although the affected patients are aware of this disturbance, they appear to ignore it and are unable to influence it. Possible associated problems include motor neglect of the contralateral side of the body or a contralateral sensory deficit. Following an acute onset, thalamic astasia usually improves over days to months [46]. The pathomechanism of the disturbance is probably related to the so-called pusher syndrome, which may occur in larger strokes: patients with marked hemiparesis or hemiplegia push away from the unaffected side due to an impaired subjective perception of the vertical in the frontal plane [47]. Lesions of the pontomesencephalic area which involve the pedunculopontine nucleus may cause marked impairment of balance and gait, with problems of gait initiation and irregular gait patterns.

### Vestibulopathic gait

Acute vestibular disturbances lead to massive insecurity when standing and walking, with a propensity to fall. Acute peripheral vestibular dysfunction on one side leads to the feeling of being pulled to the ipsilateral side and to falls to that side. Unilateral chronic vestibular dysfunction may cause consistent deviations from a straight line while walking (veering gait), which can be observed particularly well during blind gait. In patients with bilateral vestibulopathy, walking may become very insecure due to lateral pulsion, vertigo and oscillopsia while walking. These problems may be enhanced by reduced visual input, for instance in dim light or by uneven surfaces. Patients with vestibular gait disorders typically experience less difficulty while walking fast or running than during slow gait [48].

### Psychogenic gait disorders

In the past, the diagnosis of a psychogenic or functional gait disorder used to be made only once all potential organic causes had been ruled out. Nowadays, inconsistencies in the clinical neurological findings are considered the most important indicator of a functional neurological disorder. Clinical findings that support the diagnosis include a lack of persistence of a symptom or sign and temporary complete resolution while the patient feels unobserved or is being distracted by other tasks [49]. Almost any type of gait problem may occur. Patients may walk excessively slowly or show truncal trembling or jerks. Frequently, the gait appears bizarre and massively insecure. In relation to the insecurity demonstrated on examination, the patients are typically much less incapacitated in the daily lives than expected and falls are much less common. If these patients do fall, they typically do not incur injuries. Recently, effort-associated behavior disproportionate to the severity of gait impairment (the huffing and puffing sign) have been identified as highly specific for functional gait disorders [50]. Other clinical signs potentially pointing towards a functional nature of a gait disorder are manipulation-resistant dorsiflexion of the first toe, fixed plantar flexion and inversion of one foot or both feet and the “swivel chair sign”. The latter denotes a marked discrepancy between disturbed upright walking and normal forward motion when using a swivel chair [50].

Some organic neurological conditions, such as the neuroacanthocytosis syndromes or generalised dystonia, may be characterized by a bizarre walking pattern, which may lead to an erroneous diagnosis of a psychogenic gait disorder. The differential diagnoses also include cautious gait (senile gait disorder), periodic paralysis, episodic ataxias, paroxysmal dyskinesia and cataplexy. Patients with functional gait disorders should be offered physiotherapy and psychotherapy, potentially involving suggestion, although no approach has as yet been proven to be efficacious.

## Conclusion

Bipedal gait is a fundamental function that determines human life beyond early infancy almost as much as speech, higher cognitive abilities and use of complex tools. As the prevalence of gait disorders increases with age, the number of people affected will substantially increase in the coming decades due to the expected demographic changes. Gait disorders lead to a loss of personal freedom and to reduced quality of life. Gait impairments are also precursors of falls and therefore of potentially severe injuries in elderly persons.

The causes of gait disorders include neurological, orthopedic, medical and psychiatric conditions and multifactorial etiology becomes more common with advancing age, making classification and management more complex. Any gait disorder should be thoroughly investigated in order to improve patient mobility and independence, to prevent falls and to detect the underlying causes as early as possible. Thorough clinical observation of gait, careful history taking focussed on gait and falls and physical, neurological and orthopedic examinations are basic steps in the categorization of gait disorders and serve as a guide for ancillary investigations and therapeutic interventions.

Prevention and treatment of iatrogenic, especially medication-induced, gait disorders are important measures to reduce the burden of falls in the geriatric population. Several gait disorders are amenable to specific treatment. Levodopa is the drug of choice for the treatment of the gait disorder of PD and in some other parkinsonian syndromes. Rare conditions, such as myoclonus and orthostatic tremor also may respond well to medication. In normal pressure hydrocephalus, cervical spondylotic myelopathy, lumbar spinal stenosis and hip or knee osteoarthritis, surgical treatment should be considered. Patients with gait disorders not amenable to specific treatment (e. g. many neuromuscular conditions, frontal gait disorders) may benefit from multimodal rehabilitation, gait training, use of assistive devices and fall prevention measures. Commonly used exercise interventions such as muscle strength, power and resistance training as well as coordination training can improve habitual and maximum gait speed in elderly subjects [51]. These exercise programs can be individualized according to the type of gait impairment, the therapist’s experience and patient’s preferences.
